# Structural Features and Properties’ Characterization of Polylactic Acid/Natural Rubber Blends with Epoxidized Soybean Oil

**DOI:** 10.3390/polym13071101

**Published:** 2021-03-30

**Authors:** Andrey Burkov, Alexander Kraev, Maxim Grishin, Roman Vesnin, Sergey Fomin, Alexey Iordanskii

**Affiliations:** 1 Department Chemistry and Technology Polymer Processing, Vyatka State University, 610000 Kirov, Russia; usr21900@vyatsu.ru (A.K.); rl_vesnin@vyatsu.ru (R.V.); rubber@vyatsu.ru (S.F.); 2 Semenov Institute of Chemical Physics, 119991 Moscow, Russia; mvgrishin68@yandex.ru (M.G.); aljordan08@gmail.com (A.I.)

**Keywords:** polylactide, natural rubber, compatibilizer, phase structure

## Abstract

Because of the effort to preserve petroleum resources and promote the development of eco-friendly materials, bio-based polymers produced from sustainable resources have attracted great attention. Among them, polylactide (PLA) and natural rubber (NR) present prominent polymers with unique barrier and mechanical features. A series of samples with improved phase compatibility were obtained by blending PLA and NR using a double-rotor mixer. A plasticizing and enhancing effect on the polymers’ compatibility was achieved by using epoxidized soybean oil (ESO) as a natural plasticizer and compatibilizer. ESO compounding in the PLA-NR blends increased the mobility of the biopolymer’s molecular chains and improved the thermal stability of the novel material. The size of the NR domains embedded in the continuous PLA matrix decreased with the ESO content increment. The combination of thermal analysis and scanning electron microscopy enabled the authors to determine the features of potential packaging material and the optimal content of PLA-NR-ESO for the best mechanical properties.

## 1. Introduction

Polymers have firmly entered our lives as constructional and special materials, successfully competing with and replacing traditional materials, such as glass and metals. They are especially well-suited for the production of packaging materials due to their availability, relevant properties, and manufacturability. However, certain operational properties of synthetic polymers turn from being advantages to drawbacks when the polymers are recycled or scrapped. Unlike organic natural materials, polymers do not degrade for a very long time (80–100 years) and remain almost in their “initial”, stable state without decomposing, due to their resistance to chemical, atmospheric, and biological elements. Packaging materials and plastic wrappers have become quite a problem because of the huge manufacturing volumes of such products and their rather short operation life. One solution to the problem of polymer waste can and should be the manufacture of biodegradable plastics that degrade under natural conditions. Presently, one of the most common bioplastics is polylactide (PLA), a synthetic polymer of lactic acid. However, the unmodified PLA is brittle, with low impact elasticity and break elongation. Its properties are similar to those of another relatively brittle polymer—polystyrene [1,2,3]. These properties significantly limit the application of PLA in the polymer packaging manufacture. This flaw in the mechanical properties is eliminated by combining the PLA with other polymers [4] and elastomers [5,6,7,8], primarily natural rubber (NR), which is a natural biopolymer [9]. The PLA-NR based composites have been extensively studied; however, all the tests have pointed out the problem of insufficient compatibility among these polymers. A partial solution to this problem is to introduce epoxidized natural rubber [10,11,12,13] and its modifications [14,15,16], use functional modifiers in the process of reaction mixing [17,18], and pre-treat NR in order to increase its content of oxygen-containing groups [18,19]. However, all these methods require rather expensive materials.

Commercial PLA and its blends have been modified by the wide spectrum of plasticizers and compatibilizers where the synthetic chemicals dominate. However, to preserve the environment, the non-toxic and eco-friendly modifiers should be used, especially in such a delicate area of implementation as food packaging. Replacing petroleum-based plasticizers and compatibilizers with bio-based ones obtained from natural renewable resources, such as palm oil [20], epoxidized cottonseed oil [21], and limonene from the main component of citrus oil [22], could be an attractive alternative not only for environmental sustainability but also for circular economy trends [23].

This paper first covers our research of the use of epoxidized soybean oil (ESO) as a simultaneous compatibilizer and plasticizer for the PLA-unmodified NR-based compound. Epoxidized soybean oil provides excellent performance to elastomers and plastics. ESO is a non-toxic ingredient; therefore, it is used in the production of medical devices and food packaging. ESO is also a product made from renewable plant materials. In polymer compositions, ESO is characterized by low volatility and high migration resistance. The presence of a large number of oxygen-containing groups should ensure compatibility of the PLA-NR blend as the potential candidate for innovative packaging.

## 2. Materials and Methods

In our research, we used Ingeo 4043D extrusion PLA by NatureWorks (Blair, NE, USA), of 1.24 g/cm^3^ density. SMRCV60 natural rubber (Barena Group, Kuala Lumpur, Malaysia) was also used. A total of 200C epoxidized soybean oil was obtained from Galata Chemicals (Southbury, CT, USA).

Before mixing, pre-treatment of the polymers was as follows: the PLA was vacuum-dried for 8 hours at 80 °C, and NR was plasticized on a two-roll mill at ambient temperature for 15 min. This operation improves the processability of NR. The components were mixed in a closed double-rotor mixer for 30 min at 160 °C and 60 RPM. Based on the previously published recommendations [24,25] the PLA/NR ratio was 60/40. The ESO in-polymer content varied at 5, 10, 15 and 20 pph (the samples were tagged M0—no oil; M5, M10, M15, M20, respectively. The number is the ESO content). Samples for physical and mechanical tests were injection molded at 170 °C.

To run the physical and mechanical tests, the Autograph AG–X 5 kN tensile testing machine by Shimadzu (Kyoto, Japan) was used at room temperature. The upper clamp moved at 100 mm/min. All samples were tested 5 times.

The scanning electron microscopy of the obtained samples’ structure was carried out on a JSM-6510 scanning electron microscope by JEOL (Akishima City, Japan). To make the samples brittle, they were cooled in liquid nitrogen and then broken, thus giving a fresh cleavage. Following that, to improve the quality and clarity of photographs, a layer of platinum was deposited upon the surface at 4 Pa pressure. The thickness of the deposited layer was 8 nm.

Differential scanning calorimetry (DSC) was used to determine the compounds’ thermophysical properties. The DSC curves were recorded on a DSC-60 calorimeter by Shimadzu (Kyoto, Japan). The samples were heated in aluminum crucibles. The reference sample was aluminum oxide. The tests were run in a nitrogen atmosphere at 50 mL/min flow rate; the temperature range varied from room temperature to 180 °C; and the heating rate was 10 K/min. The instrument was calibrated against indium, tin, and lead. The samples inside the crucible weighed about 5 mg. To eliminate the samples’ thermal history, they were heated up to 180 °C for 5 min, cooled to room temperature, and then reheated to record the final DSC curve. All samples were tested 3 times.

The study of the thermal stability of materials was carried out on a DTG-60 synchronous thermal analyzer by Shimadzu (Kyoto, Japan). The samples were heated in platinum crucibles. The reference sample was aluminum oxide. The tests were run in an air atmosphere at 150 mL/min flow rate; the temperature range varied from room temperature to 600 °C; and the heating rate was 10 K/min. The instrument was calibrated against indium, tin, and lead. The samples inside the crucible weighed about 7 mg.

The rheological properties tests were run on the StressTech multifunctional rheometer by REOLOGICA Instruments AB (Lund, Sweden) at 170 °C. The melts’ dynamic characteristics (G’, G’’, η) were measured in the 0.01–10 Hz frequency range. The design of the test cell was that of two parallel surfaces (25 mm in work diameter).

## 3. Results and Discussion

### 3.1. Crystallization Analysis

**Figure 1:**
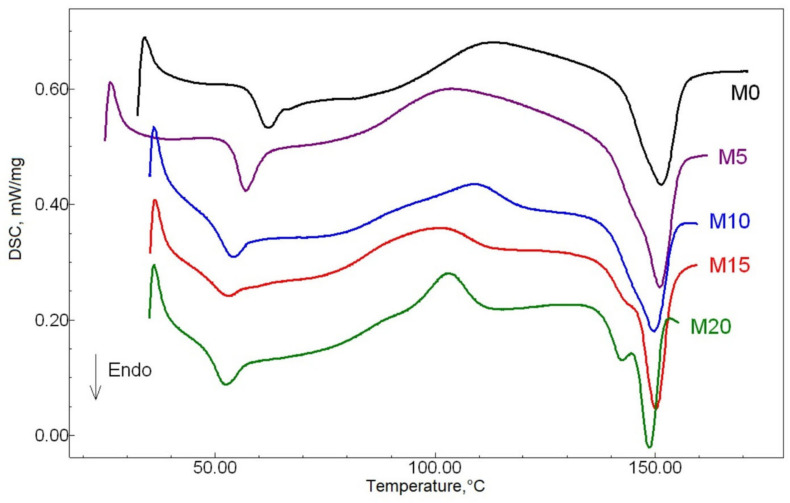
The samples’ differential scanning calorimetry (DSC) thermograms.

The DSC analysis was used to evaluate the thermophysical properties of the tested samples. Figure 1 shows DSC thermograms of the studied samples, for which the temperatures of characteristic thermal transitions and the estimated crystallinity are presented in Table 1.

Many researchers have noted the absence of a shift in the PLA glass transition temperatures when mixed with the NR [26], or that the shift is small and about 3–4 °C [27]. In our case, based on the PLA glass transition temperature, it can be concluded that the addition of ESO results in the thermoplastic phase plasticization. This is confirmed by a significant decrease in the PLA Tg from 59.4 °C (no oil) to 50.9 °C (20 mass parts of oil).

Researchers also pay close attention to the PLA’s crystallization behavior. It was demonstrated earlier [14] that the introduction of elastomers promotes greater mobility of PLA chains. In our case, the introduction of ESO additionally resulted in PLA Tc decrease. This fact can be explained by a decrease in the mobility of PLA molecules and an acceleration in the crystallization process [26]. This effect is especially pronounced in the M20 sample, where the greatest Tc shift toward low temperatures is recorded; thus, the corresponding peak is the highest of all the samples studied.

With an increase in the ESO content, the temperature of endothermic transitions corresponding to PLA melting, also shifted, as expected, towards a lower temperature range. The appearance of double peaks in M15 samples, and even more pronounced in the M20 sample, should be noted. In earlier studies, the authors [26] associated the appearance of PLA double melting peaks in the thermoplastic phase with the presence of a more ordered crystalline structure (high-temperature peak) and a less ordered, more defective structure (low-temperature peak). In our case, the appearance of a second peak at a lower melting point in the M15 and M20 samples can be explained by the formation of excessive ESO content areas, where the PLA component is characterized by a lower melting point.

The degree of crystallinity in the PLA phase decreased with an increase in the ESO content. This is due to interaction enhancement for both biopolymers that leads to the segmental mobility retardation of PLA and the reduction of crystallization ability. The analogous situation was observed for the series of PLA/poly(3-hydroxybutyrate) plasticized with PEG in the work [28].

### 3.2. Thermogravimetric Analysis

**Figure 2:**
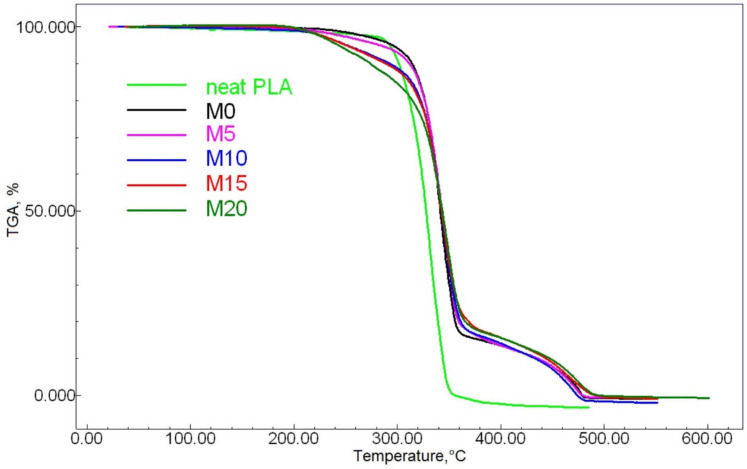
The samples’ TGA curves.

**Figure 3:**
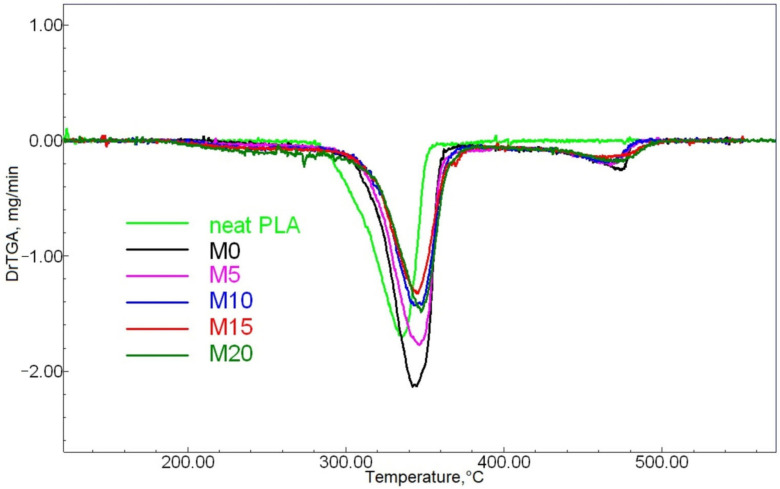
The samples’ DTG curves.

We used TGA to evaluate the ESO introduction’s effect on the analytes’ thermal stability. The TGA data are given in Figure 2 and Figure 3, while the characteristic points of mass change are shown in Table 2.

The temperature shifts at 5 and 10% mass loss points, i.e., at the initial stage of thermodestruction, toward lower temperature ranges with higher ESO content are most likely due to the volatility of this component and desorption.

The TGA curves of all of the PLA-NR mixes are clearly step-like in their mass-loss appearance, demonstrating an evident sharp change at about 360 °C. Such situations are typical for similar compounds, and this was confirmed by earlier studies of PLA mixed with either low-molecular NR [1] or epoxidized NR [14]. PLA’s thermal stability improved greatly when mixed with NR. This is proven by the shift of the 50% sample decomposition temperature by 10–14 °C. This can be explained by the higher molecular weight of NR in comparison with PLA, as well as by PLA’s very low thermal stability [29]. The introduction of ESO also had a positive effect on thermal stability: the increasing oil content caused the 50% mass loss temperature to shift by 4–6 °C toward higher values. The most significant increase in thermal stability was caused by the introduction of five mass parts of ESO; after this, the parameter did not increase so noticeably (the M5 differs from M20 by only about 2 °C). This may be due to two factors: firstly, ESO is traditionally known as a stabilizer for polymer compounds (chlorine-containing and others) with a predictable behavior; secondly, the structure of ESO molecules combining polar and nonpolar fragments, which are considerably different in nature, can act as a kind of PLA-NR compatibilizer. Thus, the introduction of ESO causes closer interfacial interaction of the studied polymers.

### 3.3. Rheological Behavior

**Figure 4:**
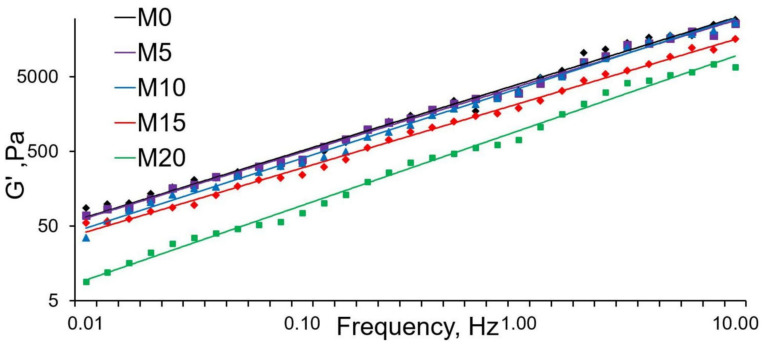
Correlation between G’ and frequency.

**Figure 5:**
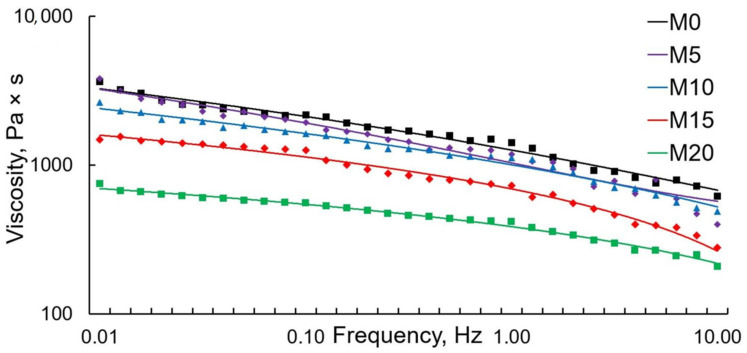
Correlation between viscosity and frequency.

The compounds’ rheological behavior is important for the estimation of material recycling through melting. Additionally, changes in the patterns of melt flow can help predict the structural features of the resulting polymer systems. The correlations between the storage modulus and dynamic viscosity are shown in Figure 4 and Figure 5.

The G’ curve of the storage modulus steadily went up as the frequency increases, which proves the previously obtained data. Abnormal behavior compared to that of the other samples is seen in sample M20—the G’ values in this case are significantly lower. The low G’ values may be due to the ESO excess amount, as part of it is no longer able to fully interact with PLA. It is also worth noting that with an increase in the ESO content, the frequency dependence of G’ became more pronounced (the graph gets steeper). This can be explained by the increase in the PLA macromolecules’ mobility.

An analysis of the complex viscosity’s relation to frequency in all samples revealed no plateau-like areas, indicating a typical non-Newtonian behavior of melts [26]. As the ESO content increased, the viscosity values dropped. This is especially noticeable in the M20 sample. This can also be explained by the excess amount of ESO, which, in this case, acts as a lubricant while the melt flows.

**Figure 6:**
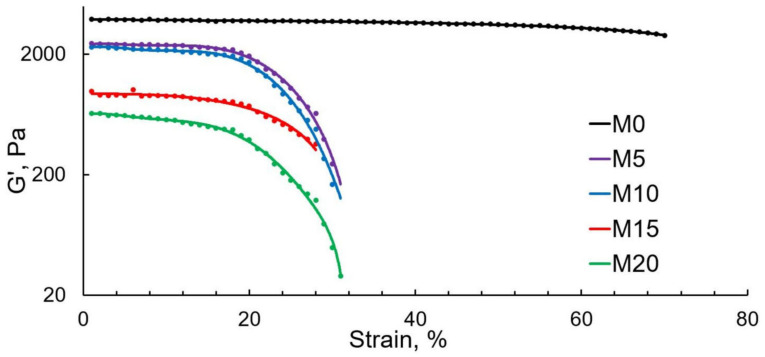
Correlation between G’ and deformation.

Figure 6 shows the relationship between the storage modulus and the applied strain. The G’ values naturally decrease as the ESO content rises; i.e., with the introduction of oil, the material becomes more viscous and less elastic. One should note the difference in the change G’ with an increase in the deformation amplitude. All ESO-containing samples demonstrated a sharp drop in G’ in the 20–40% deformation range. This can be explained by stronger PLA-NR interaction in the presence of ESO. The ESO-induced interface structures, characterized by weak interaction energy, break down almost immediately once low-amplitude deformations are applied. However, the very fact of the presence of such structures makes it possible to surmise that PLA-NR compatibility increases in the presence of ESO. This is consistent with previous studies [12,13], where it was found that epoxy groups have a positive effect on the PLA-NR compatibility. The M0 sample (no ESO) has no such structures; therefore, the relation of G’ and deformation amplitude, in this case, looks like a low-pitch (almost horizontal) curve without pronounced drops in G’ values.

**Figure 7:**
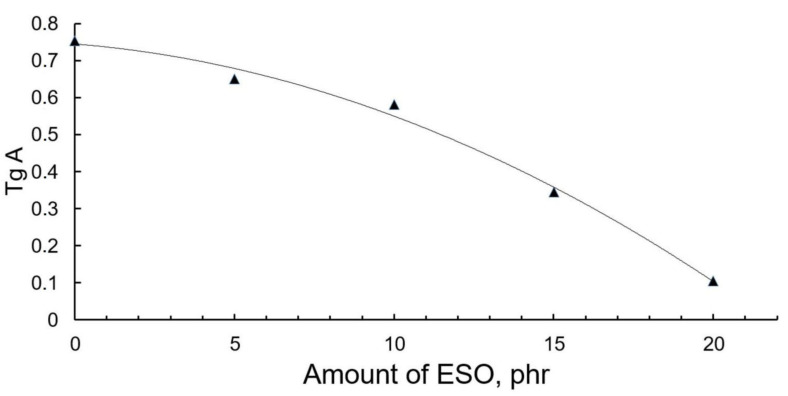
The tgA slope ratio–epoxidized soybean oil (tgA–ESO) content relationship.

The relationship plot of η″ (imaginary viscosity) and η′ (real viscosity), known as the Cole–Cole plot, is yet another tool that can be used to study the properties of polymer compounds’ structures [30]. These plots are known to take the shape of sloping curves, a semicircle, or a plateau depending on a compound’s morphology and the degree of components interaction [31]. The slope ratio (tgA) of the Cole–Cole plot in the range of small η′ values describes the degree of polymers’ compatibility. The tgA–ESO content relationship is given in Figure 7.

Higher tgA values describe greater compatibility of the mix components. The figure shows that the increase in the ESO content by more than 10 mass parts causes a much steeper tgA drop and, as a result, a greater worsening of the PLA-NR compatibility.

### 3.4. Morphology Study

**Figure 8:**
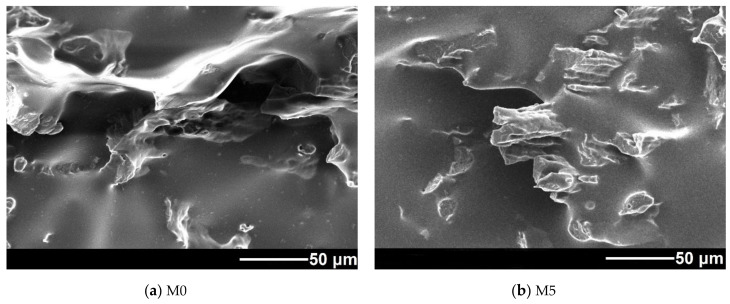

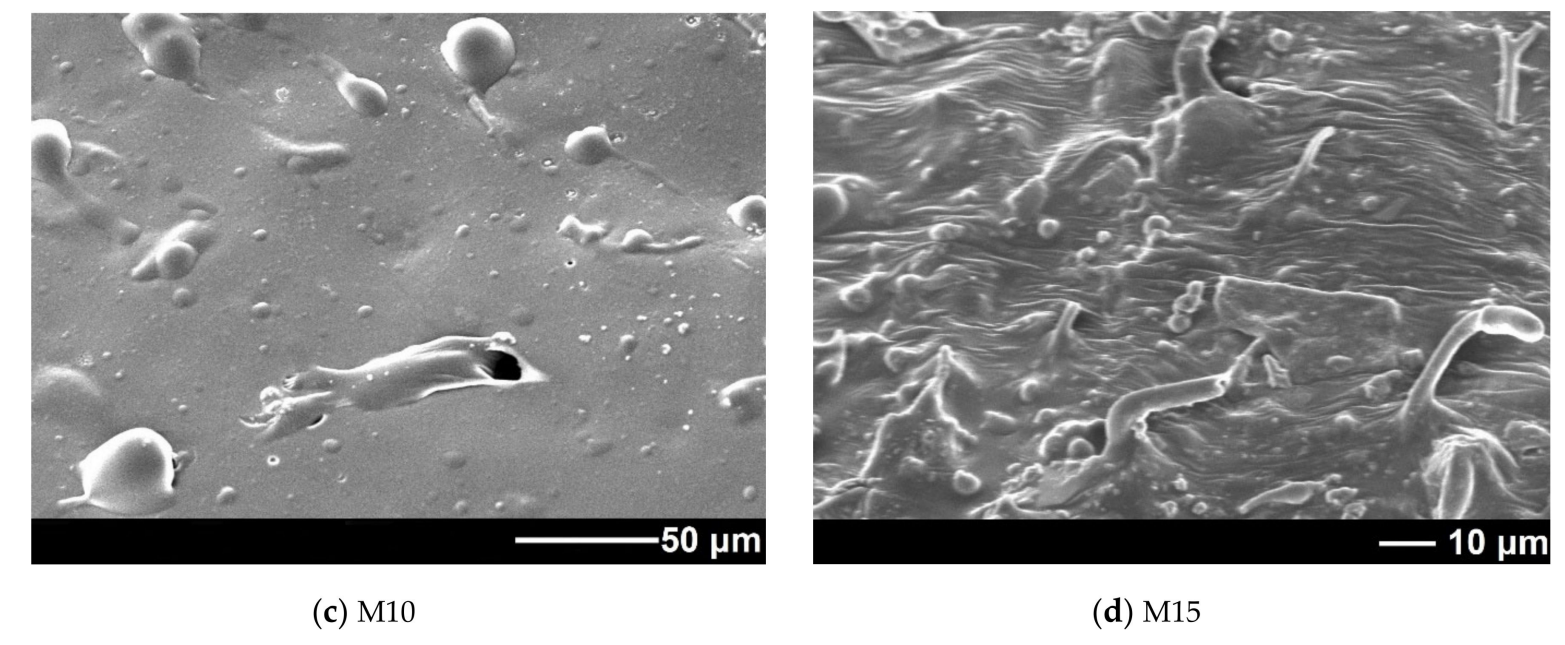
SEM images of cleavages. Samples M0 (**a**), M5 (**b**), M10 (**c**), M15 (**d**).

Microscopy is one of the most obvious and reliable methods to study the structure and morphology of polymer compounds. SEM images of the samples’ chipped surfaces are presented in Figure 8.

The image shows two incompatible polymers. By analyzing the radiation intensity distribution over the elements, it is revealed that the low-viscosity PLA makes a continuous phase where NR is distributed as separate inclusions. With the introduction of ESO, the average size of NR inclusions drops from about 10 µm (M0 sample) to a few µm (M15 sample). In comparison with the data obtained earlier [14,32], this result can be considered quite satisfactory, given the high percentage of NR. One can observe the samples’ surfaces become more homogeneous once ESO is introduced. Similar results were observed by researchers [33], who concluded that the observed results were due to improved polymer compatibility. Thus, one can confirm the efficiency of ESO as a compatibility enhancer in PLA-NR compounds. However, in our research, we failed to achieve a finer and more even distribution of polymer components or the formation of a continuous NR mesh structure, as it had been demonstrated in earlier studies utilizing other additives to the PLA-NR system (e.g., epoxidized NR [34], phenolic resin [24]).

Moreover, the images of the M15 sample register traces of greater deformations in the NR, indicating that the NR component is responsible for the deformation/elongation of the material. As was mentioned in earlier studies, this fact confirms the assumption of the improved interface interaction between NR domains and the PLA matrix. In samples with lower ESO content, rubber domains are much less deformed or not deformed at all, which negatively affects the specific elongation at break.

It should also be mentioned that SEM uncovered some heterogeneous areas with excess ESO content in the M20 sample. This confirms the assumption that the optimal oil content does not exceed 10–15 mass parts.

### 3.5. Mechanical Properties

**Figure 9:**
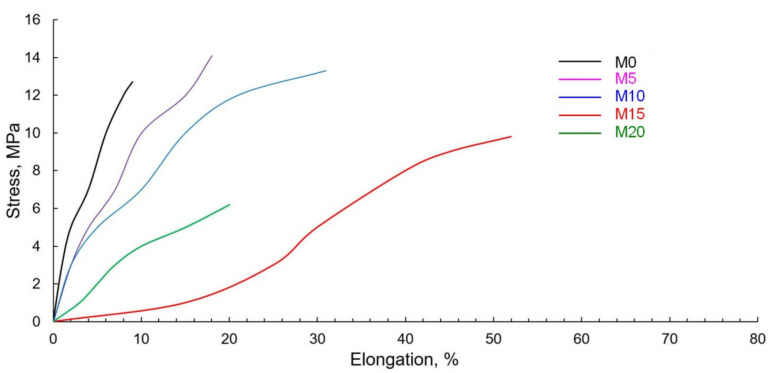
Strain–stress curves for samples with different ESO contents.

The mechanical characteristics of materials are very important for assessing the fields of their practical application. The results of mechanical tests are given in Table 3. Strain–stress curves are shown in Figure 9.

The pure PLA taken as a reference sample demonstrates the typical behavior of brittle plastic—quite large values of conventional strength and low elongation rates. With the addition of elastomer (sample M0), there is naturally a slight increase in elongation and a significant decrease in strength. The intensity of the changes in these indicators correlates with previous studies [25,35,36,37]. The introduction of 5–10 mass parts of ESO causes a slight increase in strength. This can be explained by the positive effect of epoxy groups on the PLA-NR interface interaction, which is consistent with previous studies [38]. Once the ESO content is increased to 15 and 20 mass parts, the processes of polymer component “dilution” begin prevailing. The polymer is a load-bearing component, and once it is “diluted”, the compound’s nominal tensile strength is significantly reduced. As for the specific elongation at break, the increase in this indicator to 52% with the increase in ESO content confirms the hypothesis about the role of oil as a PLA-NR compatibility enhancer. Even though the results exceed the properties of previously studied materials [39,40], they are a far cry from the compounds obtained by using the NR dynamic vulcanization [41,42]. The sharp decrease in the elongation of the M20 sample is due to the excess amount of ESO in the compound.

## 4. Conclusions

In the course of the study, a series of PLA-NR samples was produced. Objectively, the low compatibility of these polymers was improved by the introduction of ESO. The additive effectively plasticized the PLA, increased the mobility of the biopolymer’s molecular chains, and also improved the thermal stability of the material. The size of the NR domains in the continuous PLA matrix dropped to a few µm as the ESO was introduced. Through the combined use of rheological and thermal analyses, the optimal ESO amount of 5–10 mass parts was determined. In this range, the materials under study demonstrate the best mechanical properties. Thus, ESO is an effective multifunctional additive in PLA-NR blends. ESO simultaneously acts as a compatibilizer and plasticizer. As a plasticizer, ESO exhibits a decrease in viscosity, an increase in segmental mobility, processability of material, and elongation at break. Its impact as a compatibilizer upon the morphology of the blends could be proven by SEM microphotos (a decrease in the dimension for the 2nd phase). Along with these effects, ESO has the benefits related to its eco-friendly behavior [43] and bio-based origin [44]. Investigation of combinations of ESO with other compatibilizers, identification of synergistic effects of these additives, and investigation of blend morphology/dynamic mechanical characteristics by AFM and DMA can be directions for further research.

## Figures and Tables

**Table 1:** The samples’ thermal characteristics.

Samples	Tg, °C	Tc, °C	Tm, °C	X, %
M0	59.4 ± 0.1	108.6 ± 0.1	151.7 ± 0.1	8.5 ± 0.4
M5	54.9 ± 0.1	98.9 ± 0.1	151.2 ± 0.1	8.3 ± 0.4
M10	52.6 ± 0.1	109.8 ± 0.1	150.1 ± 0.1	8.2 ± 0.4
M15	51.0 ± 0.1	98.6 ± 0.1	150.2 ± 0.1	6.1 ± 0.5
M20	50.9 ± 0.1	101.8 ± 0.1	148.8 ± 0.1	6.2 ± 0.5

**Table 2:** The TGA data.

Sample	5% Mass Loss Temperatures, °C	10% Mass Loss Temperatures, °C	50% Mass Loss Temperatures, °C	90% Mass Loss Temperatures, °C
Initial PLA	293	301	328	344
M0	295	314	338	435
M5	282	311	342	435
M10	251	293	343	433
M15	250	288	343	444
M20	244	274	344	448

**Table 3:** Analytes’ mechanical test results.

	PLA	M0	M5	M10	M15	M20
τ, Mpa	55.0 ± 1.7	12.7 ± 0.6	14.1 ± 0.6	13.3 ± 0.5	9.8 ± 1.2	6.2 ± 1.9
ε, %	5 ± 1.3	9 ± 2.5	18 ± 3.9	31 ± 3.7	52 ± 7.1	20 ± 6.4

## References

[1] Bijarimi M., Ahmad S., Alam A.K.M.M. (2017). Toughening effect of liquid natural rubber on the morphology and thermo-mechanical properties of the poly (lactic acid) ternary blend. Polym. Bull..

[2] Pholharn D., Srithep Y., Morris J. (2018). Melt compounding and characterization of poly (lactide) stereocomplex/natural rubber composites. Polym. Eng. Sci..

[3] Ferri J.M., Garcia-Garcia D., Rayon E., Samper M.D., Balart R. (2020). Compatibilization and characterization of polylactide and biopolyethylene binary blends by non-reactive and reactive compatibilization approaches. Polymers.

[4] Walha F., Lamnawar K., Maazouz A., Jaziri M. (2016). Rheological, morphological and mechanical studies of sustainably sourced polymer blends based on poly(lactic acid) and polyamide 11. Polymers.

[5] Alias N.F., Ismail H., Ku Ishak K.M. (2020). Tailoring Properties of polylactic acid/rubber/kenaf biocomposites: Effects of type of rubber and kenaf loading. BioResources.

[6] Alias N.F., Ismail H., Ku Ishak K.M. (2020). Comparison of type of rubber in PLA/rubber/kenaf biocomposite: Rheological, mechanical and morphological properties. Macromol. Symp..

[7] Amran N.A.M., Ahmad S., Chen R.S., Shahdan D. (2019). Tensile properties and thermal stability of nanocomposite poly-lactic acid/liquid natural rubber filled graphene nanoplates. AIP Conf. Proc..

[8] Piontek A., Vernaez O., Kabasci S. (2020). Compatibilization of poly(lactic acid) (PLA) and bio-based ethylene-propylene-diene-rubber (EPDM) via reactive extrusion with different coagents. Polymers.

[9] Lee J.M., Hong J.S., Ahn K.H. (2019). Particle percolation in a poly (lactic acid)/calcium carbonate nanocomposite with a small amount of a secondary phase and its influence on the mechanical properties. Polym. Compos..

[10] Mustafa S.N.I.S., Man S.H.C., Hassan A., Baharulrazi N. (2020). Enhancement of toughness and thermal properties of polylactic acid/liquid epoxidized natural rubber/graphene oxide composites. AIP Conf. Proc..

[11] Klinkajorn J., Tanrattanakul V. (2020). The effect of epoxide content on compatibility of poly (lactic acid)/epoxidized natural rubber blends. J. Appl. Polym. Sci..

[12] Yamamoto Y., Ishida T., Kosugi K., Kawahara S., Nghia P.T. (2018). Crystallization behavior and mechanical property of epoxidized natural rubber/poly (lactic acid) blend. Kgk Kautsch. Gummi Kunstst..

[13] Mongkolvai A., Chuayjuligit S., Chaiwutthinan P., Larpkasemsuk A., Boonmahitthisud A. (2018). Preparation and properties of poly (lactic acid)/epoxidized natural rubber/nano-silica composites. Key Eng. Mater..

[14] Tessanan W., Chanthateyanonth R., Yamaguchi M., Phinyocheep P. (2020). Improvement of mechanical and impact performance of poly (lactic acid) by renewable modified natural rubber. J. Clean. Prod..

[15] Sathornluck S., Choochottiros C. (2019). Modification of epoxidized natural rubber as a PLA toughening agent. J. Appl. Polym. Sci..

[16] Sookprasert P., Hinchiranan N. (2017). Morphology, mechanical and thermal properties of poly (lactic acid)(PLA)/natural rubber (NR) blends compatibilized by NR-graft-PLA. J. Mater. Res..

[17] Huang J., Fan J., Yuan D., Zhang S., Chen Y. (2020). Facile preparation of supertoughened polylactide-based thermoplastic vulcanizates without sacrificing the stiffness based on the selective distribution of silica. Ind. Eng. Chem. Res..

[18] Zhao X., Hu H., Wang X., Yu X., Zhou W., Peng S. (2020). Super tough poly (lactic acid) blends: A comprehensive review. RSC Adv..

[19] Phetphaisit C.W., Wapanyakul W., Phinyocheep P. (2019). Effect of modified rubber powder on the morphology and thermal and mechanical properties of blown poly (lactic acid)–hydroxyl epoxidized natural rubber films for flexible film packaging. J. Appl. Polym. Sci..

[20] Ali F.B., Awale R.G., Fakhruldin H., Anuar H. (2016). Plasticizing poly (lactic acid) using epoxidized palm oil for environmental friendly packaging material. Malays. J. Anal. Sci..

[21] Carbonell-Verdu A., Samper R.D., Garcia-Garcia D., Sanchez-Nacher L., Balart R. (2017). Plasticization effect of epoxidized cottonseed oil (ECSO) on poly (lactic acid). Ind. Crop. Prod..

[22] Arrieta M.P., Lopez J., Ferrandiz S., Peltzer M.A. (2013). Characterization of PLA-limonene blends for food packaging applications. Polym. Test..

[23] Geueke B., Groh K., Muncke J. (2018). Food packaging in the circular economy: Overview of chemical safety aspects for commonly used materials. J. Clean. Prod..

[24] Yuan D., Chen Z., Chen K., Mou W., Chen Y. (2016). Phenolic resin-induced dynamically vulcanized polylactide/natural rubber blends. Polym. Plast. Technol. Eng..

[25] Wang W., Huang J., Gong Z., Fan J., Cao L., Chen Y. (2020). Biobased PLA/NR-PMMA TPV with balanced stiffness-toughness: In-situ interfacial compatibilization, performance and toughening model. Polym. Test..

[26] Si W.J., Yuan W., Li Y., Chen Y., Zeng J. (2018). Tailoring toughness of fully biobased poly (lactic acid)/natural rubber blends through dynamic vulcanization. Polym. Test..

[27] Mustafa S.N.I.S., Man S.H.C., Baharulrazi N., Mohamad Z., Hassan A., Yusof N.H. (2020). Mechanical and thermal properties of polylactic acid/liquid epoxidized natural rubber blends. Chem. Eng. Trans..

[28] Rogovina S., Zhorina L., Gatin A., Prut E., Kuznetsova O., Yakhina A., Olkhov A., Samoylov N., Grishin M., Iordanskii A. (2020). Biodegradable polylactide-poly(3-Hydroxybutyrate) compositions obtained via blending under shear deformations and electrospinning. Characterization and environmental application. Polymers.

[29] Rosli N.A., Ahmad I., Anuar F.H., Abdullah I. (2018). The contribution of eco-friendly bio-based blends on enhancing the thermal stability and biodegradability of poly (lactic acid). J. Clean. Prod..

[30] Bitinis N., Verdejo R., Cassagnau P., Lopez-Monchado M.A. (2011). Structure and properties of polylactide/natural rubber blends. Mater. Chem. Phys..

[31] Salehiyan R., Ray S.S., Stadler F.G., Ojijo V. (2018). Rheology–microstructure relationships in melt-processed polylactide/poly (vinylidene fluoride) blends. Materials.

[32] Tábi T., Hajba S. (2019). Cross effect of natural rubber and annealing on the properties of poly(lactic acid). Periodica Polytech. Mech. Eng..

[33] Mohammad N.N.B., Arsad A., Sani N.A., Basri M.H. (2017). Effect of compatibilisers on thermal and morphological properties of polylactic acid/natural rubber blends. Chem. Eng. Trans..

[34] Abdullah N.A.S., Mohamad Z., Man S.H.C., Baharulrazi N., Majid R.A., Jusoh M., Ngadi N. (2019). Thermal and toughness enhancement of poly (lactic acid) bio-nanocomposites. Chem. Eng. Trans..

[35] Xia M., Lang W., Yang Y., Yu J., Wu N., Wang Q. (2019). The microstructure of GNR and the mechanical properties of biobased PLA/GNR thermoplastic vulcanizates with excellent toughness. Materials.

[36] Mohamad Z., Syahida N.N.N., Othman N., Baharulrazi N., Man S.H.C., Jusoh M., Majid R.A. (2018). Properties enhancement of polylactic acid biocomposite. Chem. Eng. Trans..

[37] Liu Y., Cao L., Yuan D., Chen Y. (2018). Design of super-tough co-continuous PLA/NR/SiO_2_ TPVs with balanced stiffness-toughness based on reinforced rubber and interfacial compatibilization. Compos. Sci. Technol..

[38] Yuan D., Ding J., Mou W., Wang Y., Chen Y. (2017). Bio-based polylactide/epoxidized natural rubber thermoplastic vulcanizates with a co-continuous phase structure. Polym. Test..

[39] Buys Y.F., Aznan A.N.A., Anuar H. (2018). Mechanical properties, morphology, and hydrolytic degradation behavior of polylactic acid/natural rubber blends. IOP Conf. Ser. Mater. Sci. Eng..

[40] Shahdan D., Chen R.S., Ahmad S., Zailan F.D., Ali A.M. (2018). Assessment of mechanical performance, thermal stability and water resistance of novel conductive poly (lactic acid)/modified natural rubber blends with low loading of polyaniline. Polym. Int..

[41] Huang J., Mou W., Wang W., Lv F., Chen Y. (2020). Influence of DCP content on the toughness and morphology of fully biobased ternary PLA/NR-PMMA/NR TPVs with co-continuous phase structure. Polym. Plast. Technol. Mater..

[42] Phattarateera S., Pattamaprom C. (2019). The effect of dynamic vulcanization on the mechanical and thermal properties of stereocomplex PLLA/PDLA/rubber blends. IOP Conf. Ser. Mater. Sci. Eng..

[43] Agnihotri S., Shukla S., Pradeep S.A., Pilla S. (2019). Biobased thermosetting cellular blends: Exploiting the ecological advantage of epoxidized soybean oil in structural foams. Polymer.

[44] Sahoo K., Mohanty S., Nayak S.K. (2015). Synthesis and characterization of bio-based epoxy blends from renewable resource based epoxidized soybean oil as reactive diluent. Chin. J. Polym. Sci..

